# Development and validation of a multidimensional student health assessment scale for primary and secondary school students

**DOI:** 10.3389/fpsyg.2026.1850546

**Published:** 2026-07-17

**Authors:** Yanling Li, Da Deng

**Affiliations:** 1 School of Education, Geely University of China, Chengdu, China; 2 School of Teacher Education, Chengdu University, Chengdu, China

**Keywords:** healthy lifestyle, holistic student health, physical activity literacy, psychosocial competence, relational-contextual support, school health assessment

## Abstract

**Background:**

Student health is increasingly understood as a multidimensional construct shaped by both individual functioning and contextual support. However, existing school-based measures often focus on single domains and provide limited coverage of holistic health in primary and secondary school populations.

**Objective:**

This study aimed to develop and validate the Multidimensional Student Health Assessment Scale for Primary and Secondary School Students.

**Methods:**

A multi-stage psychometric design was used to develop and validate the Multidimensional Student Health Assessment Scale for Primary and Secondary School Students. Item generation was guided by multidimensional health theory, ecological perspectives, and the Chinese holistic health perspective, followed by expert review for content validity. Five independent samples were used: 385 students for pilot testing and item analysis, 400 for exploratory factor analysis, 1,014 for confirmatory factor analysis and construct validity evaluation, 87 for test–retest reliability, and 3,818 for preliminary known-groups validation. Reliability and construct validity were evaluated using internal consistency, composite reliability, average variance extracted, discriminant validity, temporal stability, and preliminary known-groups evidence. Additional analyses included parallel analysis, measurement invariance testing across educational stages and gender, and McDonald's omega.

**Results:**

A four-factor structure was retained, comprising Relational-Contextual Support and Prosocial Orientation, Health Resource and Physical Activity Literacy, Healthy Lifestyle, and Psychosocial Competence. The four-factor model showed acceptable CFA fit and adequate internal consistency. However, parallel analysis suggested a more conservative factor solution, and educational-stage comparisons were therefore interpreted cautiously. Test–retest reliability over a 3-week interval was acceptable. Known-groups analyses showed systematic differences across educational stages and self-reported vision-status groups, providing preliminary known-groups evidence.

**Conclusions:**

The scale provides initial psychometric evidence for group-level assessment of holistic student health. However, further validation using external criterion measures, robust estimators, alternative structural models, and developmentally sensitive methods is needed.

## 1 Introduction

Health in children and adolescents is increasingly understood as a multidimensional and developmentally embedded construct that extends beyond the absence of disease. Rather than referring only to physical condition, student health is now widely interpreted as involving psychological functioning, social relationships, and the broader contexts in which young people grow and learn (World Health Organization, 2026, 2021, World Health Organization, United Nations Educational, Scientific and Cultural Organization, 2021, Bronfenbrenner and Morris, 2006). This broader perspective is particularly important in school-aged populations because health-related capacities and vulnerabilities formed during childhood and adolescence can shape later academic engagement, psychosocial adaptation, and long-term wellbeing (Bronfenbrenner and Morris, 2006).

Recent international frameworks have further emphasized that schools are not merely educational institutions, but key developmental settings for promoting child and adolescent health. The World Health Organization (WHO) guideline on school health services highlights that school health provision should address students' physical, mental, social, and developmental needs in an integrated way (World Health Organization, 2021). Likewise, the WHO/UNESCO Global Standards for Health-Promoting Schools advocate whole-school approaches in which school climate, participation, relationships, and supportive environments are treated as central components of health promotion World Health Organization, United Nations Educational, Scientific and Cultural Organization, 2021. Recent international scholarship has also called for broader and more integrated approaches to adolescent health and wellbeing that can respond to both long-standing and emerging developmental challenges (Baird et al., 2025; Innocenti UNICEF – Global Office of Research Foresight, 2025).

In China, the need for multidimensional assessment of student health has become increasingly salient under the combined influence of the Healthy China agenda, educational reform, and recent school health governance initiatives. In particular, the Action Plan for Comprehensively Strengthening and Improving Student Mental Health in the New Era (2023–2025), jointly issued by 17 governmental departments including the Ministry of Education, emphasizes coordinated school–family–society efforts to promote students' healthy development (Ministry of Education of the People's Republic of China, 2023). More recently, national policy has further highlighted the “health first” orientation and the construction of healthier school environments as part of the broader effort to improve student wellbeing (Ministry of Education of the People's Republic of China, 2026).

However, measurement practice has not fully kept pace with this conceptual and policy shift. Existing instruments used in schools and research often focus on specific domains, such as physical fitness, mental health symptoms, health literacy, or quality of life (Jorm, 2012; Paakkari and Paakkari, 2012; Ravens-Sieberer et al., 2007). Although these tools provide valuable information, they also tend to generate fragmented evidence that is insufficient for evaluating students' holistic health functioning in educational contexts. This limitation is especially evident when moral/social functioning and environmental support are theoretically recognized as relevant to child and adolescent development, yet are rarely incorporated as core components within a unified and psychometrically tested framework (Bronfenbrenner and Morris, 2006; Eccles et al., 1993).

Against this background, the Chinese “Big Health” perspective offers a potentially useful extension by emphasizing whole-person, whole-process, and context-sensitive health development (Lei, 2016; Yan et al., 2017; Shen and Zeng, 2020). Conceptually, this perspective is compatible with ecological systems theory, which stresses that child development unfolds through reciprocal interactions between individuals and their surrounding environments (Bronfenbrenner and Morris, 2006; Bronfenbrenner, 1979). It also resonates with recent international discussions of child and adolescent wellbeing, which increasingly frame health as the product of both internal capacities and contextual supports (Baird et al., 2025; Innocenti UNICEF – Global Office of Research Foresight, 2025).

In the present study, holistic student health is defined as a multidimensional developmental state in which students' physical functioning, health-related behaviors, psychological and psychosocial competence, moral/social development, and perceived environmental support interact to shape their capacity for healthy learning and living. This definition emphasizes that student health is not located solely within the individual but is co-constructed through everyday interactions among personal behaviors, emotional and social functioning, moral orientation, family and school support, and broader ecological conditions. Therefore, an assessment of holistic student health should incorporate physical health, psychological functioning, moral/social development, and environmental support in tandem rather than treating them as isolated domains.

Despite growing theoretical and policy attention to holistic student health, a psychometrically validated multidimensional instrument specifically designed for primary and secondary school students remains lacking. In particular, few available measures simultaneously operationalize physical health, psychological functioning, moral/social development, and environmental support within a single measurement model suitable for school-based research and group-level monitoring. Although the initial conceptual framework was theory-driven, the present study treated scale development as an iterative process in which the empirical factor structure could refine the original theoretical assumptions. Therefore, the final factor labels and interpretation were determined by integrating the initial multidimensional health framework with the observed item clustering from exploratory and confirmatory analyses.

## 2 Methods

### 2.1 Study design

This study used a multi-stage psychometric design to develop and validate a multidimensional instrument for assessing holistic health among primary and secondary school students. The design was guided by established principles of scale development and construct validation and aimed to generate evidence for content validity, internal structure, reliability, and external validity (DeVellis, 2016; Clark and Watson, 1995; American Educational Research Association, 2014; Boateng et al., 2018).

To reduce capitalization on chance and strengthen the credibility of the factor structure, separate samples were used for pilot testing, exploratory factor analysis (EFA), confirmatory factor analysis (CFA), test–retest reliability, and known-groups validation (Brown, 2015; Kline, 2015). The validation process included four sequential stages: (1) item generation and refinement based on theory, prior literature, and expert review; (2) pilot testing and item analysis; (3) exploration of the latent structure using EFA; and (4) evaluation of structural validity and additional reliability and validity evidence using CFA and external analyses, with five independent samples allocated across these stages.

### 2.2 Participants and sampling procedure

Participants were recruited from primary schools, junior high schools, and senior high schools in Sichuan Province, China, using a multi-stage cluster sampling approach. The sampling process was designed to cover different educational stages and school contexts. First, cooperating schools were identified through collaboration with local education authorities. Second, schools from primary, junior high, and senior high levels were invited to participate. Third, intact classes within participating schools were selected and surveyed to preserve natural classroom structures and reduce selection bias.

The five samples were collected for different validation purposes and were treated as independent samples across the scale development process. Sample 1 was used for pilot testing and item analysis, Sample 2 for exploratory factor analysis, Sample 3 for confirmatory factor analysis and construct validity evaluation, Sample 4 for test–retest reliability, and Sample 5 for preliminary known-groups validation. Participants were not intentionally reused across exploratory and confirmatory validation stages, thereby reducing capitalization on chance.

Sample size adequacy was considered according to the analytic purpose of each stage. For item analysis and pilot testing, Sample 1 provided sufficient cases for preliminary item evaluation. For EFA, Sample 2 included 400 valid responses, which exceeded commonly recommended participant-to-item ratios for factor analysis. For CFA, Sample 3 included 1,014 valid responses, which was sufficient for structural equation modeling of a model with moderate complexity. Sample 5 included 3,818 valid responses, providing sufficient statistical power for group comparisons and measurement invariance testing across educational stages and gender.

**Sample 1: Pilot testing and item analysis**. Sample 1 was used for pilot testing, item discrimination analysis, and preliminary internal consistency assessment. A total of 385 students were recruited from one administrative district, including 130 primary school students, 123 junior high school students, and 132 senior high school students. The sample included 190 boys (49.4%) and 195 girls (50.6%).

**Sample 2: Exploratory factor analysis**. Sample 2 was used for EFA. Questionnaires were distributed to students from 27 schools through an online survey platform. A total of 432 responses were collected, and 400 valid responses were retained after excluding patterned responses and questionnaires completed in implausibly short times, yielding a valid response rate of 92.6%. The sample size was adequate for factor analysis relative to the number of candidate items (DeVellis, 2016; Brown, 2015).

**Sample 3: Confirmatory factor analysis**. Sample 3 was used for CFA and construct validity evaluation. This sample was independent of Sample 2 and included 1,014 valid responses collected using similar procedures. The sample size was sufficient for structural equation modeling of a model with moderate complexity (Brown, 2015; Kline, 2015).

**Sample 4: Test–retest reliability**. Sample 4 was used to evaluate temporal stability. One hundred students were invited using stratified sampling to ensure balanced representation across educational stages and gender, and 87 students completed both administrations at a 3-week interval (response rate = 87%).

**Sample 5: Known-groups validation**. Sample 5 was used to assess known-groups validity. A total of 3,900 questionnaires were distributed across 78 schools, and 3,818 valid responses were returned (response rate = 97.9%). The sample included 1,988 boys (52%) and 1,830 girls (48%), as well as 2,374 primary school students, 855 junior high school students, and 589 senior high school students.

The large sample size provided sufficient statistical power to detect group differences across educational stages and vision status categories.

Inclusion criteria were: enrollment in a participating primary, junior high, or senior high school; ability to understand the questionnaire instructions; provision of student assent; and written informed consent from parents or legal guardians. Exclusion criteria included missing parental consent or student assent, incomplete questionnaires, patterned responses, duplicate submissions, and questionnaires completed in implausibly short times. All students who met the eligibility criteria in the selected classes were invited to participate, and no eligible student was excluded on the basis of academic performance, gender, boarding status, or health status.

The educational stages examined broadly corresponded to late childhood and adolescence. In the Chinese school system, primary school generally covers Grades 1–6, junior high school covers Grades 7–9, and senior high school covers Grades 10–12, corresponding approximately to ages 6–12, 12–15, and 15–18 years, respectively. Because exact age information was not available for all participants, educational stage was used as the primary developmental grouping variable.

### 2.3 Item generation and instrument development

Item generation followed a theory-driven approach grounded in international health frameworks and the Chinese holistic health perspective. Consistent with recommendations for construct development, the initial item pool was derived from a clearly specified conceptual definition of student health as a multidimensional construct integrating individual functioning and contextual support (DeVellis, 2016; Clark and Watson, 1995).

Three sources informed the development of the construct framework. First, multidimensional views of health and ecological models of development were used to conceptualize student health as shaped by interactions between the individual and surrounding systems. Second, school health and health-promotion perspectives supported the inclusion of both personal competencies and environmental conditions. Third, prior work on health literacy, mental health literacy, psychosocial functioning, and school climate informed the identification of candidate content domains and representative indicators (Jorm, 2012; Paakkari and Paakkari, 2012; Ravens-Sieberer et al., 2007; Eccles et al., 1993).

Based on this conceptual synthesis, student health was initially specified as a four-dimensional construct comprising: (1) physical health, including health knowledge, behavioral patterns, and functional competence; (2) psychological health, including awareness, emotional regulation, interpersonal functioning, and active health promotion; (3) moral/social health, including moral cognition, moral emotion, moral will, and moral behavior; and (4) environmental health, including living, school, family, and broader social environments.

These four domains served as the initial theoretical framework for item generation rather than as a fixed measurement structure. The final factor structure was determined empirically through EFA and then evaluated using CFA, reliability analysis, and additional validity evidence.

These domains were conceptualized as correlated first-order latent constructs reflecting distinct but interrelated components of holistic student health. All subjective items were designed as reflective indicators and were rated on a five-point Likert scale ranging from 1 (strongly disagree) to 5 (strongly agree). An initial pool of 33 items was generated to ensure adequate coverage across all domains (see Table 1). Item wording was refined to improve conceptual clarity, age appropriateness, and readability for primary and secondary school students.

**Table 1:** Initial conceptual framework of multidimensional student health.

Domain	Conceptual components	Item codes
Physical health	Health knowledge and awareness	A1–A10
	Health behavioral patterns	
	Health skills and functional competence	
Psychological health	Psychological awareness	B1–B7
	Emotional regulation	
	Interpersonal psychological functioning	
	Psychological health promotion	
Moral/social health	Moral cognition	C1–C9
	Moral emotion	
	Moral will	
	Moral behavior	
Environmental health	Living environment	D1–D5
	School environment	
	Family environment	
	Social environment	

**Figure 1:**
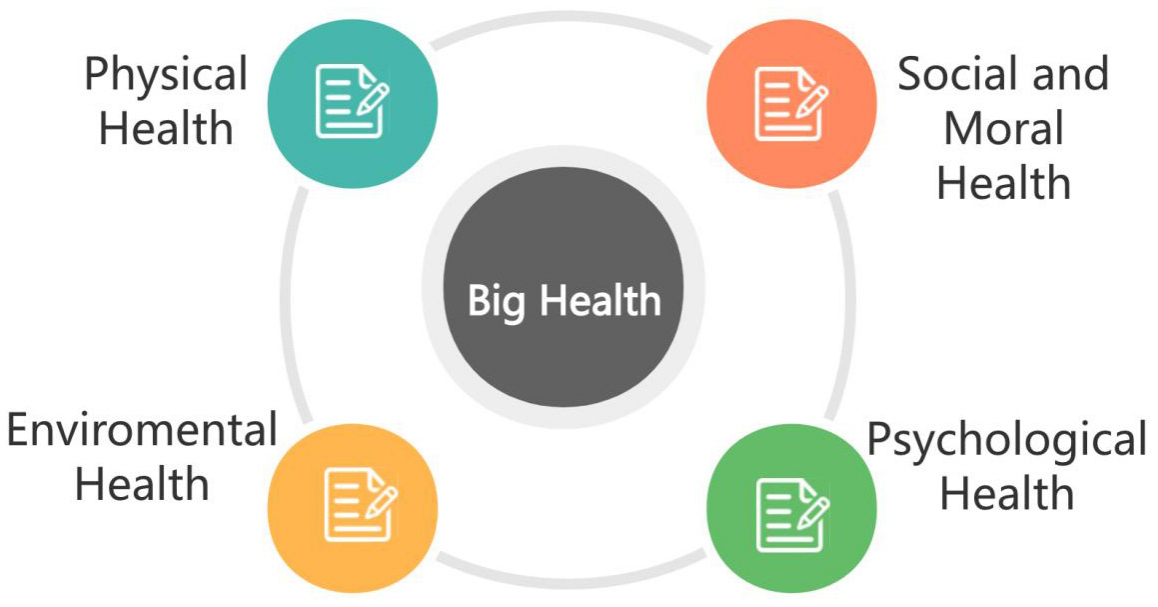
Conceptual framework of the multidimensional student health scale.

Two single-item self-reported health-status variables (A9 and A10), representing vision status and perceived body-shape status, were retained for descriptive and known-groups analyses but were not treated as reflective indicators of latent constructs. Accordingly, they were excluded from factor-analytic procedures (DeVellis, 2016). The conceptual framework of the multidimensional student health construct is presented in Figure 1, and the complete questionnaire is provided in Appendix A.

### 2.4 Content validity evaluation

Content validity was evaluated through expert review to determine whether the preliminary items were relevant to and representative of the intended construct domain. In line with current measurement standards, content validity refers to the degree to which the content of an instrument adequately reflects the construct it is intended to measure (American Educational Research Association, 2014; Polit and Beck, 2006; Polit et al., 2007).

Six experts with doctoral-level training and research experience in educational psychology, health education, and psychometrics independently reviewed the preliminary questionnaire. All experts had prior experience in scale development and child or adolescent health research. Each reflective item was rated on a four-point relevance scale ranging from 1 (not relevant) to 4 (highly relevant), and ratings of 3 or 4 were treated as indicating acceptable relevance.

For each item, the item-level content validity index (I-CVI) was calculated as the proportion of experts assigning a relevance rating of 3 or 4. For a six-expert panel, an I-CVI of 0.78 or above was treated as acceptable (Polit and Beck, 2006). Scale-level content validity was evaluated using the average method (S-CVI/Ave), with values of 0.80 or above indicating satisfactory overall content representativeness (Polit et al., 2007). To account for possible chance agreement, modified kappa coefficients (k^*^) were also calculated and interpreted using established criteria (Polit et al., 2007).

The two single-item self-reported health-status variables (A9 and A10) were excluded from content validity calculations because they were treated as descriptive variables rather than reflective indicators. As shown in Table 2, the I-CVI values for the reflective items ranged from 0.67 to 1.00. Most items met or exceeded the recommended threshold, indicating satisfactory content relevance. Three items (A8, B6, and C8) yielded I-CVI values of 0.67 and modified kappa values of 0.569, suggesting fair but suboptimal agreement beyond chance; these items were therefore revised for clarity and age appropriateness before pilot testing. The pre- and post-revision wording of these items is presented in Table 3. The remaining items were retained because expert feedback supported their conceptual relevance within the proposed framework.

**Table 2:** Expert content validity (*n* = 6).

Item	I-CVI	Modified kappa (k^*^)	Decision
A1	1	1	Retained
A2	1	1	Retained
A3	0.83	0.816	Retained
A4	1	1	Retained
A5	1	1	Retained
A6	0.83	0.816	Retained
A7	0.83	0.816	Retained
A8	0.67	0.565	Revised
B1	1	1	Retained
B2	0.83	0.816	Retained
B3	0.83	0.816	Retained
B4	1	1	Retained
B5	1	1	Retained
B6	0.67	0.565	Revised
B7	1	1	Retained
C1	1	1	Retained
C2	1	1	Retained
C3	0.83	0.816	Retained
C4	1	1	Retained
C5	1	1	Retained
C6	0.83	0.816	Retained
C7	0.83	0.816	Retained
C8	0.67	0.565	Revised
C9	1	1	Retained
D1	1	1	Retained
D2	0.83	0.816	Retained
D3	1	1	Retained
D4	1	1	Retained
D5	0.83	0.816	Retained

**Table 3:** Revision of items after expert review.

Item	Original wording	Revised wording	Reason for revision
A8	I know basic methods of managing minor sports injuries.	I understand basic methods for managing minor sports injuries.	Improved clarity and age appropriateness
B6	I am familiar with existing psychological support resources.	I am aware of available psychological support resources.	
C8	I maintain ethical behavior without external supervision.	I behave ethically even without external supervision.	

Overall indices are presented in Table 4. The scale-level content validity index based on the average method (S-CVI/Ave) was 0.908, indicating satisfactory overall content representativeness. Modified kappa coefficients ranged from 0.565 to 1.00, suggesting fair-to-excellent agreement beyond chance across items. Taken together, these findings support the content validity of the preliminary instrument and justified proceeding to pilot testing and empirical item evaluation. The detailed expert rating matrix is provided in the Appendix B.

**Table 4:** Overall indices.

I-CVI range	0.67–1.00
Mean I-CVI (S-CVI/Ave)	0.908
k^*^ range	0.565–1.00

### 2.5 Procedure and ethical considerations

The study was approved by the Academic Ethics Committee of Geely University of China. All procedures complied with the Declaration of Helsinki and relevant national requirements for research involving minors.

Data collection was conducted in cooperating primary and secondary schools after institutional permission had been obtained from school administrators. Questionnaires were administered in classroom settings under the supervision of trained research assistants. Because all participants were younger than 18 years, written informed consent was obtained from parents or legal guardians, and assent was obtained from the students themselves before participation. Participation was voluntary, and students were informed that they could withdraw at any time without penalty or academic consequences.

No personally identifiable information was collected. Student identification numbers were used only for response matching across study phases, such as the test–retest administration, and were removed before analysis. All data were stored securely and were accessible only to the research team.

### 2.6 Item analysis

Item analysis was conducted using Sample 1 to evaluate item discrimination and preliminary internal consistency before factor analysis. Following established recommendations for scale development, item performance was examined using both extreme-group comparisons and corrected item–total correlations (DeVellis, 2016).

First, the extreme-group method was applied by comparing the upper and lower 27% of participants ranked by total scale scores. Independent-samples *t*-tests were performed for each item. Second, corrected item–total correlations were calculated to evaluate the relationship between each item and the overall scale score. Items showing weak discrimination or weak item–total associations were considered for exclusion or revision. Corrected item–total correlations of 0.30 or above were treated as acceptable (DeVellis, 2016).

The two single-item self-reported health-status variables (A9 and A10) were not treated as reflective items and therefore were not retained for subsequent factor-analytic procedures. Preliminary internal consistency was also examined using Cronbach's alpha.

### 2.7 Exploratory factor analysis

Exploratory factor analysis (EFA) was conducted using Sample 2 to examine the latent structure of the reflective items. Before factor extraction, sampling adequacy and factorability of the correlation matrix were assessed using the Kaiser–Meyer–Olkin (KMO) measure and Bartlett's test of sphericity (Kaiser, 1974).

Principal axis factoring was used as the extraction method because the purpose of the analysis was to identify latent common factors rather than maximize total variance (Fabrigar et al., 1999). Given the theoretical expectation that dimensions of student health would be correlated, an oblique rotation method (direct oblimin) was applied (Fabrigar et al., 1999).

Factor retention was determined by considering eigenvalues, scree plot inspection, cumulative variance explained, theoretical interpretability, and parallel analysis (DeVellis, 2016; Fabrigar et al., 1999). To further evaluate the factor-retention decision, parallel analysis was conducted using the Sample 2 EFA dataset. The observed eigenvalues were compared with eigenvalues generated from random datasets of the same size. Because reliance on a single retention rule may produce unstable solutions, substantive interpretability was considered alongside statistical criteria. The retained factor solution was then taken forward to confirmatory factor analysis.

### 2.8 Confirmatory factor analysis

Confirmatory factor analysis (CFA) was conducted using Sample 3 to test the factor structure identified in the EFA. Structural equation modeling was performed in AMOS using maximum likelihood estimation. Maximum likelihood estimation was used because the items were measured on five-point Likert scales and the CFA sample size was large. Five-category Likert-type items are often treated as approximately continuous in applied CFA when distributional assumptions are not severely violated. Nevertheless, because the items were ordinal in nature, the use of maximum likelihood estimation is acknowledged as a methodological limitation, and future studies should examine the robustness of the model using ordinal or robust estimators. The hypothesized model specified correlated latent factors, with each observed item loading only on its designated factor. No cross-loadings were specified, and residual covariances were not estimated unless theoretically justified.

Model fit was evaluated using multiple complementary indices, including the chi-square statistic (χ^2^), degrees of freedom (df), the chi-square to degrees-of-freedom ratio (χ^2^/df), the Comparative Fit Index (CFI), Tucker–Lewis Index (TLI), Incremental Fit Index (IFI), Root Mean Square Error of Approximation (RMSEA), and Standardized Root Mean Square Residual (SRMR) (Brown, 2015; Kline, 2015; Hu and Bentler, 1999). In line with widely used guidelines, χ^2^/df values below 3, CFI/TLI/IFI values of 0.90 or above, and RMSEA/SRMR values below 0.08 were taken to indicate acceptable model fit (Kline, 2015; Hu and Bentler, 1999). Standardized factor loadings were examined to assess the extent to which each item represented its intended latent construct.

### 2.9 Reliability and validity

Several forms of reliability and construct validity evidence were examined following the CFA. Internal consistency reliability was first assessed using Cronbach's alpha for each retained subscale. In addition, composite reliability (CR) was calculated from standardized factor loadings and error variances derived from the CFA model because CR provides a construct-level reliability estimate that is less sensitive than alpha to the number of items in a scale (Fornell and Larcker, 1981). Values of 0.70 or above were considered acceptable.

Convergent validity was evaluated using the average variance extracted (AVE), which reflects the proportion of variance captured by a latent construct relative to measurement error (Fornell and Larcker, 1981). AVE values of 0.50 or above were interpreted as indicating adequate convergent validity.

Discriminant validity was examined using the Fornell–Larcker criterion, according to which the square root of the AVE for each construct should exceed its correlations with other constructs (Fornell and Larcker, 1981).

McDonald's omega was additionally calculated using the Sample 3 CFA dataset to supplement Cronbach's alpha, because omega provides a less restrictive estimate of internal consistency when tau-equivalence cannot be assumed.

### 2.10 Common method bias

Because all reflective items were collected through self-report questionnaires, common method variance was examined using Harman's single-factor test as a preliminary diagnostic procedure. An unrotated exploratory factor analysis was conducted to determine whether a single factor accounted for the majority of the total variance. If one general factor explained more than 50% of the variance, common method variance would be considered a potentially serious concern (Podsakoff et al., 2003). This procedure was used as a preliminary diagnostic rather than a definitive test of method effects.

### 2.11 Test–retest reliability

Temporal stability was evaluated using Sample 4, in which participants completed the questionnaire twice over a 3-week interval. Test–retest reliability was estimated using the intraclass correlation coefficient (ICC) based on a two-way random-effects model with absolute agreement for single measures, that is, ICC(2,1) (Koo and Li, 2016). This form of ICC is appropriate when the aim is to assess absolute agreement across repeated administrations. Ninety-five percent confidence intervals were also reported.

ICC values were interpreted using commonly applied criteria proposed by Cicchetti (1994), with values below 0.40 indicating poor reliability, 0.40–0.59 fair reliability, 0.60–0.74 good reliability, and 0.75–1.00 excellent reliability.

### 2.12 Preliminary known-groups evidence

Preliminary known-groups evidence was examined using educational stage and self-reported vision status as grouping variables by using Sample 5. These analyses were intended to provide initial evidence of whether the scale was sensitive to theoretically relevant group differences, rather than to establish full criterion-related validity.

Group differences in total scores and subscale scores were analyzed using one-way analysis of variance (ANOVA). When omnibus group effects were statistically significant, *post-hoc* comparisons were conducted using Bonferroni correction to control for Type I error. Effect sizes were estimated using eta-squared (η^2^), with values of 0.01, 0.06, and 0.14 conventionally interpreted as small, medium, and large, respectively (Cohen, 1988). Statistical significance was evaluated at p < 0.05.

### 2.13 Measurement invariance

Measurement invariance across educational stages and gender was examined using multi-group confirmatory factor analysis. Configural, metric, and scalar invariance models were tested sequentially. Configural invariance evaluated whether the same factor structure was supported across groups. Metric invariance constrained factor loadings to equality across groups, and scalar invariance further constrained item thresholds to equality. Invariance was evaluated primarily based on changes in fit indices, including ΔCFI, ΔRMSEA, and ΔSRMR. Values of ΔCFI ≤ 0.010, ΔRMSEA ≤ 0.015, ΔSRMR ≤ 0.030 for metric invariance, and ΔSRMR ≤ 0.010 for scalar invariance were considered supportive of invariance.

## 3 Results

### 3.1 Item analysis and reliability

Item analysis was conducted using Sample 1 (*N* = 385). As shown in Table 4, all retained reflective items showed significant discrimination between the upper and lower 27% groups (*p* < 0.001), with corrected item–total correlations ranging from 0.58 to 0.84, indicating adequate to strong item discrimination. In contrast, the two single-item self-reported health-status variables (A9 and A10) showed weak discrimination and low or negative correlations with the total score and were therefore excluded from subsequent factor-analytic procedures.

Preliminary internal consistency was high. The overall Cronbach's alpha exceeded 0.96, and alpha-if-item-deleted values ranged from 0.963 to 0.969, indicating that deletion of any retained reflective item would not materially improve internal consistency. Together, these findings supported retention of the reflective items for exploratory factor analysis (Table 5).

**Table 5:** Item discrimination, item–total correlations, and internal consistency reliability of the pilot scale.

Item	Total correlation (*r*)	|CR| value (*t*)	Alpha coefficient after deletion of item	Recommendation
A1	0.629	7.968^***^	0.9647	Retained
A2	0.582	7.009^***^	0.9649	Retained
A3	0.586	8.723^***^	0.9653	Retained
A4	0.631	9.842^***^	0.9648	Retained
A5	0.621	7.793^***^	0.9647	Retained
A6	0.592	6.274^***^	0.9651	Retained
A7	0.714	10.510^***^	0.9641	Retained
A8	0.69	9.084^***^	0.9643	Retained
A9	−0.153	1.995^*^	0.969	Remove
A10	−0.045	1.702	0.9675	Remove
B1	0.804	11.648^***^	0.9635	Retained
B2	0.724	9.462^***^	0.964	Retained
B3	0.769	10.544^***^	0.9637	Retained
B4	0.785	13.401^***^	0.9636	Retained
B5	0.78	11.951^***^	0.9636	Retained
B6	0.671	8.030^***^	0.9644	Retained
B7	0.798	14.012^***^	0.9636	Retained
C1	0.782	11.331^***^	0.9637	Retained
C2	0.772	11.866^***^	0.9638	Retained
C3	0.793	12.298^***^	0.9636	Retained
C4	0.819	13.415^***^	0.9634	Retained
C5	0.692	10.212^***^	0.9643	Retained
C6	0.721	10.796^***^	0.9641	Retained
C7	0.72	11.173^***^	0.9641	Retained
C8	0.754	11.598^***^	0.9639	Retained
C9	0.788	14.920^***^	0.9636	Retained
D1	0.749	13.442^***^	0.9639	Retained
D2	0.791	13.239^***^	0.9636	Retained
D3	0.774	14.449^***^	0.9637	Retained
D4	0.835	15.292^***^	0.9634	Retained
D5	0.731	10.923^***^	0.964	Retained

CR, critical ratio.

^***^p < 0.001, ^*^p < 0.05.

### 3.2 Exploratory factor analysis

EFA was performed on Sample 2 (*N* = 400). The data were suitable for factor analysis, with an excellent KMO value of 0.963 and a significant Bartlett's test of sphericity, χ^2^(406) = 8,431.02, *p* < 0.001. A four-factor solution was retained. As summarized in Tables 5–9, this solution accounted for 64.94% of the total variance. Item communalities ranged from 0.546 to 0.728, and primary factor loadings ranged from 0.401 to 0.863 in absolute value.

The retained factors were interpreted as: F1 Relational-Contextual Support and Prosocial Orientation (12 items), F2 Health Resource and Physical Activity Literacy (four items), F3 Healthy Lifestyle (five items), and F4 Psychosocial Competence (eight items)Negative loadings observed for F4 reflected factor orientation and did not alter substantive interpretation. Overall, the EFA results supported a four-factor representation of the construct (Tables 6–10).

**Table 6:** Factor 1: relational-contextual support and prosocial orientation (12 items).

Item	Factor loading	Communality
C2	0.450	0.728
C5	0.651	0.623
C6	0.687	0.629
C7	0.798	0.684
C8	0.551	0.600
C9	0.636	0.695
D1	0.532	0.664
D2	0.618	0.642
D3	0.863	0.727
D4	0.605	0.717
D5	0.694	0.578
B7	0.401	0.614

**Table 7:** Factor 2: health resource and physical activity literacy (four items).

Item	Factor loading	Communality
A3	0.544	0.546
A7	0.603	0.683
A8	0.646	0.711
B6	0.469	0.567

**Table 8:** Factor 3: healthy lifestyle (five items).

Item	Factor loading	Communality
A1	0.694	0.638
A2	0.698	0.559
A4	0.653	0.604
A5	0.556	0.595
A6	0.572	0.565

**Table 9:** Factor 4: psychosocial competence (eight items).

Item	Factor loading	Communality
B1	−0.531	0.629
B2	−0.702	0.697
B3	−0.725	0.674
B4	−0.715	0.687
B5	−0.713	0.707
C1	−0.564	0.704
C3	−0.527	0.692
C4	−0.501	0.674

**Table 10:** Eigenvalues and explained variance.

Factor	Eigenvalue	Variance explained (%)	Cumulative variance (%)
Factor 1	14.820	51.105	51.105
Factor 2	1.826	6.297	57.402
Factor 3	1.207	4.162	61.565
Factor 4	0.979	3.376	64.941

It should be noted that the empirical factor structure did not fully reproduce the original theoretically specified domains. In particular, B7 loaded with the relational and contextual support items, suggesting that general life satisfaction may be closely associated with students' perceived social, moral, family, school, and community support. B6 loaded with the health literacy and physical activity-related items, which may indicate that students interpreted awareness of psychological support resources as part of broader health-resource literacy. Therefore, the factor labels were refined to better reflect the empirical item clustering.

Additional parallel analysis suggested a more conservative factor solution (Table 11). Specifically, only the first two observed eigenvalues exceeded the corresponding random eigenvalues based on the 95th percentile criterion. Therefore, the four-factor solution was not supported by parallel analysis alone. However, the four-factor solution was retained for subsequent CFA because it provided clearer theoretical interpretability, differentiated key domains of holistic student health, and was further evaluated through CFA, reliability analysis, and measurement invariance testing. The retention of the fourth factor is, therefore, interpreted cautiously.

**Table 11:** Parallel analysis for factor retention.

Factor	Observed eigenvalue	Mean random eigenvalue	95th percentile random eigenvalue	Decision
1	14.821	1.534	1.609	Retain
2	1.826	1.458	1.511	Retain
3	1.207	1.401	1.446	Do not retain
4	0.979	1.353	1.394	Do not retain
5	0.878	1.309	1.345	Do not retain
6	0.719	1.269	1.303	Do not retain

### 3.3 Internal consistency reliability

Internal consistency reliability of the final scale was examined using Sample 3. As shown in Table 12, Cronbach's alpha and McDonald's omega coefficients indicated adequate internal consistency. The total-scale omega was 0.915, and omega coefficients for the four subscales ranged from 0.840 to 0.927.

**Table 12:** Internal consistency reliability of the final scale.

Subscale	Construct	Number of items	Cronbach's α	McDonald's ω
Total scale		29	0.915	0.915
F1	Relational-contextual support and prosocial orientation	12	0.927	0.927
F2	Health resource and physical activity literacy	4	0.838	0.84
F3	Healthy lifestyle	5	0.844	0.845
F4	Psychosocial competence	8	0.908	0.908

In addition to Cronbach's alpha, McDonald's omega was calculated. The total-scale omega was 0.915, and omega coefficients for the four subscales ranged from 0.840 to 0.927. These results provided additional evidence of adequate internal consistency reliability.

### 3.4 Confirmatory factor analysis

CFA was conducted using Sample 3 (*N* = 1,014) to test the four-factor model identified in the EFA. As shown in Table 13, the model demonstrated good fit to the data: χ^2^(371) = 1,015.95, χ^2^/df = 2.738, CFI = 0.956, TLI = 0.952, IFI = 0.956, RMSEA = 0.041 (90% CI [0.038, 0.044]), and SRMR = 0.024.

**Table 13:** Confirmatory factor analysis of the subscales (*N* = 1,014).

Indicator	Criteria	Actual fitted value
χ^2^ (CMIN)	–	1,015.95
df	–	371
χ^2^/df (CMIN/DF)	< 3	2.738
GFI	>0.9	0.936
AGFI	>0.9	0.925
RMSEA	< 0.08	0.041
CFI	>0.9	0.956
TLI (NNFI)	>0.9	0.952
IFI	>0.9	0.956
PGFI	>0.5	0.798
PNFI	>0.5	0.853
PCFI	>0.5	0.874

**Figure 2:**
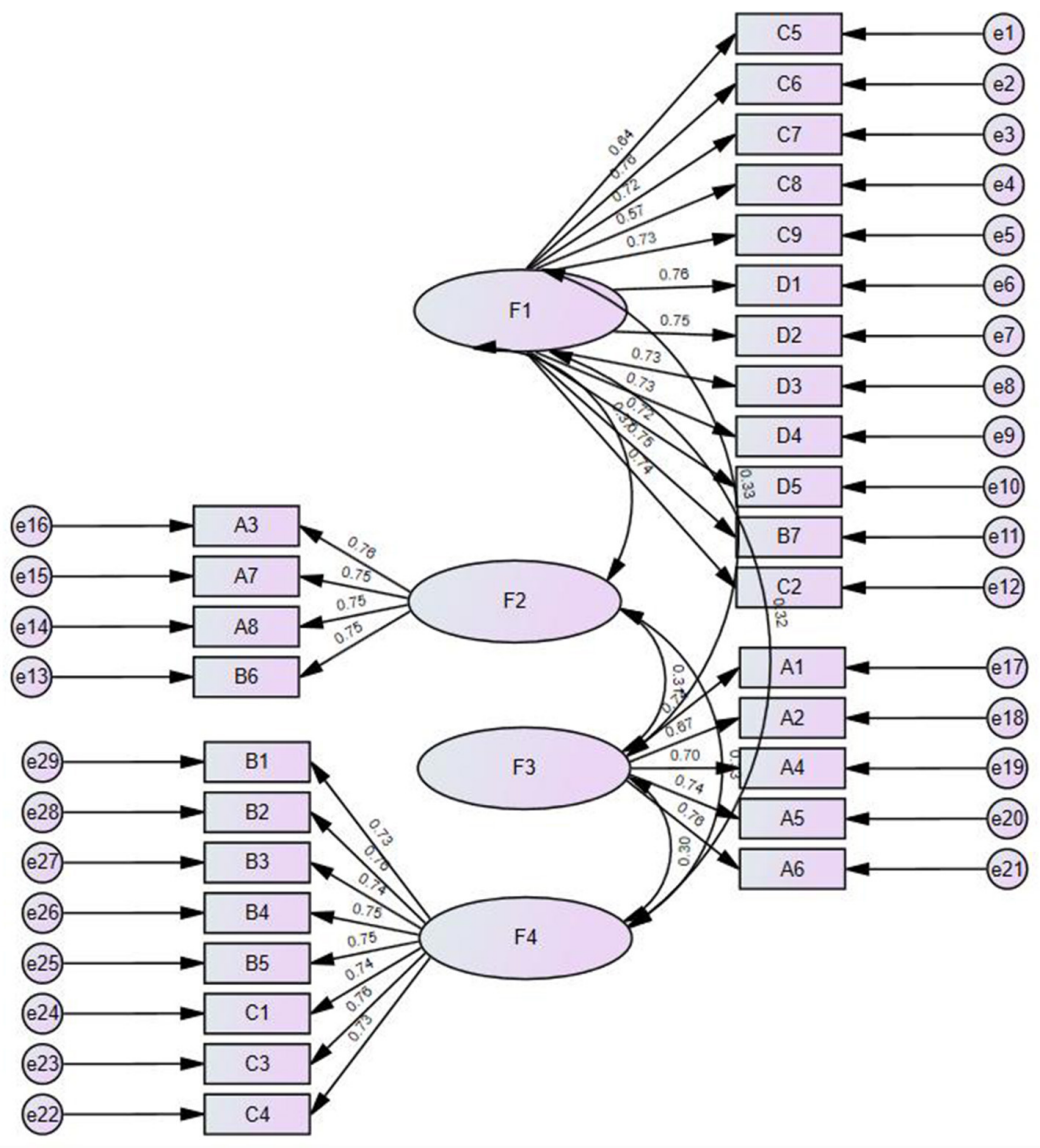
Confirmatory factor analysis.

All standardized factor loadings were statistically significant (*p* < 0.001). As illustrated in Figure 2, each observed indicator loaded appropriately on its intended latent construct. Inter-factor correlations were moderate, supporting related but distinguishable dimensions within the measurement model. Taken together, the CFA findings provided confirmatory support for the theoretically informed four-factor model, although the factor-retention results from parallel analysis suggest that the structure should be interpreted cautiously (Table 13; Figure 2).

### 3.5 Convergent validity

Convergent validity results are presented in Table 14. Composite reliability values ranged from 0.840 to 0.927, and AVE values ranged from 0.516 to 0.567 across the four factors. All CR values exceeded 0.70 and all AVE values exceeded 0.50, supporting adequate convergent validity for each construct.

**Table 14:** Convergent validity (*N* = 1,014).

Construct	Number of items	CR	AVE
F1	12	0.927	0.516
F2	4	0.84	0.567
F3	5	0.845	0.521
F4	8	0.908	0.553

### 3.6 Discriminant validity

As shown in Table 15, the square roots of AVE ranged from 0.718 to 0.753 and exceeded the corresponding inter-factor correlations, which ranged from 0.297 to 0.369. This pattern supported discriminant validity and indicated that each factor shared more variance with its own indicators than with the other constructs in the model.

**Table 15:** Discriminant validity based on the Fornell–Larcker criterion (*N* = 1,014).

Construct	F1	F2	F3	F4
F1	0.718	0.369	0.327	0.325
F2	0.369	0.753	0.31	0.333
F3	0.327	0.31	0.722	0.297
F4	0.325	0.333	0.297	0.744

### 3.7 Common method bias

The first unrotated factor accounted for 34.6% of the total variance, which was below the conventional 50% threshold. This result suggested that a single dominant method factor was not evident. However, because Harman's single-factor test is only a preliminary diagnostic, common method variance cannot be completely ruled out.

### 3.8 Test–retest reliability

Temporal stability was examined in Sample 4 (*N* = 87). As shown in Table 16, the total scale yielded an ICC of 0.607 (95% CI [0.456, 0.725], *p* < 0.001). Subscale ICCs ranged from 0.639 to 0.748, with all coefficients statistically significant (*p* < 0.001). These results indicated acceptable temporal stability over the 3-week interval.

**Table 16:** Test–retest reliability (*N* = 87).

Dimension	Intraclass correlation coefficient (ICC)	Standard
Total	0.607	Good
F1	0.742	Good (close to excellent)
F2	0.639	Good
F3	0.748	Good (close to excellent)
F4	0.743	Good (close to excellent)

### 3.9 Measurement invariance

Measurement invariance across educational stages was examined using Sample 5. The configural model showed acceptable RMSEA and SRMR values, although the CFI was slightly below 0.90. The metric and scalar models showed changes in fit indices within commonly used thresholds. Specifically, the change in CFI was −0.004 from the configural to metric model and −0.006 from the metric to scalar model, while RMSEA remained unchanged and SRMR changes were within acceptable limits. These results supported approximate measurement invariance across primary, junior high, and senior high school students. However, because the absolute CFI values were slightly below 0.90, educational-stage comparisons were interpreted cautiously (Table 17).

**Table 17:** Measurement invariance across educational stages.

Model	χ^2^	df	CFI	TLI	RMSEA	SRMR	ΔCFI	ΔRMSEA	ΔSRMR
Configural	11,479.08	1,113	0.894	0.884	0.049	0.048	—	—	—
Metric	11,914.11	1,163	0.89	0.885	0.049	0.067	−0.004	0	0.02
Scalar	12,518.41	1,213	0.884	0.884	0.049	0.065	−0.006	0	−0.002

Measurement invariance across gender was also supported. The configural, metric, and scalar models showed acceptable fit, and changes in CFI, RMSEA, and SRMR were within recommended thresholds. These findings suggested that the scale functioned similarly for boys and girls (Table 18).

**Table 18:** Measurement invariance across gender.

Model	χ^2^	df	CFI	TLI	RMSEA	SRMR	ΔCFI	ΔRMSEA	ΔSRMR
Configural	10,279.79	742	0.905	0.896	0.058	0.041	—	—	—
Metric	10,505.69	767	0.903	0.898	0.058	0.052	−0.002	0	0.011
Scalar	10,636.09	792	0.902	0.9	0.057	0.052	−0.001	−0.001	0

### 3.10 Preliminary known-groups evidence

Known-groups validity was examined in Sample 5 (*N* = 3,818), with results summarized in Table 19. Significant differences were observed across educational stages for the total score, *F*_(2, 3, 815)_ = 103.40, *p* < 0.001, η^2^ = 0.051. Mean total scores showed a monotonic gradient, with primary school students scoring highest, followed by junior high school students and then senior high school students. Significant differences were also found for all four subscales, with the largest effect observed for F1 Relational-Contextual Support and Prosocial Orientation, η^2^ = 0.072. Bonferroni-adjusted *post-hoc* comparisons indicated that all pairwise contrasts were significant.

**Table 19:** Preliminary known-groups evidence.

Variable	Group	*N*	Mean (SD)	*F*	*p*	η^2^
Educational stage	
Total	Primary	2,374	132.46 (15.95)	103.4	< 0.001	0.051
	Junior high	855	129.54 (18.72)			
	Senior high	589	120.93 (20.93)			
F1	Primary	2,374	56.29 (5.90)	147.17	< 0.001	0.072
F2	Primary	2,374	16.95 (3.48)	28.46	< 0.001	0.015
F3	Primary	2,374	22.61 (3.25)	72.28	< 0.001	0.037
F4	Primary	2,374	36.61 (4.91)	92.77	< 0.001	0.046
Vision status	
Total	No nearsightedness	1,855	133.81 (15.09)	73.16	< 0.001	0.054
	Mild	1,218	128.61 (18.71)			
	Moderate	661	123.02 (20.02)			
	Severe	84	122.12 (23.19)			
F1	No nearsightedness	1,855	56.64 (5.65)	79.4	< 0.001	0.059
F2	No nearsightedness	1,855	17.33 (3.30)	30.62	< 0.001	0.024
F3	No nearsightedness	1,855	22.85 (3.07)	50.41	< 0.001	0.038
F4	No nearsightedness	1,855	36.99 (4.65)	72.7	< 0.001	0.054

A similar graded pattern was observed across vision-status groups. Total scores differed significantly by vision status, *F*_(3, 3, 814)_ = 73.16, *p* < 0.001, and η^2^ = 0.054, with the highest scores among students without nearsightedness and progressively lower scores across mild, moderate, and severe nearsightedness groups. Comparable trends were evident across subscales, particularly for F1 and F4. Overall, these findings provided preliminary known-groups evidence. Because approximate measurement invariance across educational stages was supported based on changes in fit indices but the absolute CFI values were slightly below 0.90, educational-stage differences should be interpreted cautiously. In addition, because established external criterion instruments were not included, these results should not be interpreted as definitive evidence of criterion-related validity (Table 19).

## 4 Discussion

### 4.1 Theoretical implications

The present study developed and validated a multidimensional student health assessment scale for primary and secondary school students. Overall, the findings support the view that student health can be meaningfully represented as a multidimensional construct that includes behavioral, psychosocial, moral/social, and environmental components. Rather than reducing health to physical status or mental health symptoms alone, the retained factor structure suggests that holistic student health is more appropriately understood as an integrated profile of individual functioning and contextual support. This interpretation is broadly consistent with ecological perspectives on child development and with current school-health frameworks that emphasize the interdependence of students and their everyday environments (World Health Organization, 2021; World Health Organization, United Nations Educational, Scientific and Cultural Organization, 2021, Bronfenbrenner and Morris, 2006; Baird et al., 2025; Innocenti UNICEF – Global Office of Research Foresight, 2025; Bronfenbrenner, 1979).

One important finding was that moral/social and environmental items converged into a broader relational-contextual factor. This finding should not be interpreted as definitive evidence that moral/social development and environmental support are indistinguishable constructs. Rather, it suggests that, in students' self-reports, prosocial orientation, perceived support, and contextual experiences may be closely intertwined. At the same time, this convergence may also reflect overlapping item wording, shared school-context framing, or insufficient conceptual differentiation among the initial item domains. Therefore, this factor should be interpreted cautiously and requires further validation in future studies.

The unexpected placement of B6 and B7 further suggests that some items may have been interpreted differently by students than originally intended. B7 may reflect students' overall appraisal of their relational and environmental experiences, whereas B6 may represent broader health-resource awareness rather than psychological functioning alone. These findings highlight the need for further item refinement and alternative model testing.

Another theoretically relevant finding was the observed difference across educational stages. Primary school students scored higher than junior high school students, who in turn scored higher than senior high school students. Although the observed effect sizes were not large, the consistency of this pattern across total and subscale scores suggests that the scale may have captured developmentally relevant variation rather than isolated statistical differences. This pattern is broadly consistent with prior work suggesting that adolescence and more demanding school environments may be associated with increased academic stress, more complex social demands, and greater challenges to wellbeing (Baird et al., 2025; Innocenti UNICEF – Global Office of Research Foresight, 2025; Eccles et al., 1993). However, because the present study was cross-sectional, these differences should not be interpreted causally.

Taken together, these findings suggest that the proposed multidimensional student health framework may offer a useful conceptual bridge between Chinese holistic health discourse and international multidimensional approaches to child and adolescent wellbeing. The present study does not establish a definitive model of student health; rather, it provides initial empirical support for a multidimensional and contextually embedded representation that can be examined further in future research.

### 4.2 Measurement contribution

This study makes several methodological contributions to the assessment of student health. First, the scale was developed and evaluated through a multi-stage psychometric process that included expert review, pilot testing, EFA, CFA, internal consistency analysis, test–retest reliability, and known-groups validation. The use of separate samples for exploratory and confirmatory stages strengthened the credibility of the retained factor structure and reduced the likelihood that the findings were driven by sample-specific overfitting (DeVellis, 2016; American Educational Research Association, 2014; Boateng et al., 2018; Brown, 2015; Kline, 2015; Polit and Beck, 2006; Polit et al., 2007).

Second, the study provided multiple forms of validity and reliability evidence rather than relying on a single indicator. In addition to Cronbach's alpha, composite reliability and McDonald's omega were examined to provide complementary estimates of internal consistency. Construct validity was evaluated through both convergent and discriminant evidence, and measurement invariance across educational stages and gender was additionally tested to strengthen the interpretation of group comparisons. Temporal stability and preliminary known-groups evidence further extended the evidential basis of the instrument beyond internal structure alone. In this respect, the study moves beyond narrow item-screening approaches and contributes a more comprehensive validation strategy for a school-based health assessment tool.

Third, the study offers an empirically tested instrument that attempts to integrate domains that are often measured separately in school-based research, especially psychosocial functioning, moral/social orientation, and perceived environmental support. This integrative feature may be particularly useful in educational contexts where student outcomes are shaped by both individual and contextual conditions. At the same time, the present evidence should be understood as an initial validation foundation rather than a complete psychometric account. Further work is still needed to examine additional properties, including stronger forms of invariance across broader demographic groups, alternative structural models, robust ordinal estimators, and relationships with external criterion measures.

### 4.3 Practical implications

The findings also have practical relevance for school-based assessment and student-support systems. First, the multidimensional structure of the scale suggests that schools may benefit from moving beyond narrow assessments of physical indicators or psychological symptoms when evaluating student health. By incorporating health-related behaviors, psychosocial competence, prosocial orientation, and relational-contextual support, the instrument provides a broader profile of student functioning that may help identify areas of relative strength and vulnerability at the group level.

Second, the observed differences across educational stages suggest that the scale may be useful for identifying group-level patterns that warrant further attention during school transitions. However, these differences should be interpreted cautiously. Although approximate measurement invariance across educational stages was supported based on changes in fit indices, the absolute CFI values were slightly below 0.90. Therefore, the observed educational-stage differences should be regarded as preliminary rather than conclusive evidence of developmental variation. Used appropriately, the scale may help schools identify grade-level or stage-level patterns that require further investigation and support. For younger students, the scale should be administered with clear instructions and, when necessary, teacher clarification to ensure comprehension without guiding responses.

Third, the graded differences observed across self-reported vision-status groups suggest that the scale may be sensitive to health-relevant background conditions. However, because vision status was self-reported rather than clinically assessed, these findings should be interpreted as preliminary known-groups evidence rather than objective clinical validation. The scale is not intended for clinical diagnosis or individual-level medical decision making. Its primary value lies in group-level assessment, program evaluation, and research on holistic student health in school contexts.

### 4.4 Limitations and future directions

Several limitations should be acknowledged. First, although the four-factor model showed acceptable CFA fit and was theoretically interpretable, parallel analysis suggested a more conservative factor solution. Therefore, the retained four-factor structure should be regarded as a theoretically informed and empirically acceptable preliminary model rather than a definitive factor structure. Future studies should compare alternative models, including two-factor, three-factor, higher-order, and bifactor structures, using independent samples.

Second, although measurement invariance across gender was supported and approximate measurement invariance across educational stages was supported based on changes in fit indices, the absolute CFI values for educational-stage invariance were slightly below 0.90. Therefore, comparisons across educational stages should be interpreted cautiously and should not be treated as conclusive evidence of developmental differences.

Third, temporal stability of the total scale was acceptable but not high. This may partly reflect the fact that student health, as conceptualized here, includes context-sensitive and developmentally dynamic aspects rather than purely stable traits. Nonetheless, additional longitudinal work over longer follow-up intervals is needed to clarify the stability and responsiveness of the instrument over time.

Fourth, common method variance was examined only using Harman's single-factor test, which is a limited diagnostic procedure. Future studies should incorporate stronger procedural and statistical approaches, such as marker variables, multi-informant data, or latent method factor models.

Fifth, the CFA was estimated using maximum likelihood with five-point Likert-type items. Although this approach is common in applied research with large samples, ordinal or robust estimators such as WLSMV may provide more appropriate estimates for ordered categorical data. Future studies should re-examine the model using robust estimation methods.

Sixth, the present study did not include established external criterion measures, such as health literacy, quality of life, school climate, or psychological wellbeing instruments. Therefore, criterion-related validity remains insufficiently examined. Future studies should correlate the scale with validated external measures to determine whether it adequately captures holistic student health.

Seventh, the use of the same questionnaire across primary, junior high, and senior high school students requires further evidence. Students at different developmental stages may differ in their comprehension of abstract items related to psychological resources, moral judgment, and environmental support. Future studies should conduct cognitive interviews, readability analyses, or differential item functioning analyses and consider developing stage-specific or short-form versions of the scale.

Finally, the study was conducted in Sichuan Province, China, and the findings should therefore be generalized with caution. Replication in other regions and cultural contexts would help determine the broader applicability of the scale. Despite these limitations, the present study provides an initial empirical basis for the assessment of holistic student health and offers a foundation for further refinement and cross-context validation.

## 5 Conclusion

This study developed and initially validated a multidimensional student health assessment scale for primary and secondary school students. The findings provide preliminary evidence that student health can be assessed through a multidimensional framework integrating health behaviors, psychosocial competence, prosocial orientation, and contextual support. The four-factor structure demonstrated acceptable CFA fit, reliability, and partial support for measurement invariance; however, parallel analysis suggested a more conservative structure, and external criterion-related validity remains to be examined. Therefore, the scale should be regarded as an initial tool for group-level assessment and research rather than a fully established measure. Further studies are needed to test alternative models, external criterion validity, developmental appropriateness, and short-form versions across broader populations.

## Data Availability

The datasets presented in this study are available in an online repository at: https://pan.baidu.com/s/1592WiuZ7taXnuQ2W_XMGXA. Access code: b6rx.
