# Supplementary material for: Development and validation of a multidimensional student health assessment scale for primary and secondary school students
Source: Front Psychol. 2026 Jul 17;17:1850546. doi: 10.3389/fpsyg.2026.1850546 (PMC13425453; doi:10.3389/fpsyg.2026.1850546)
Supplement: Supplementary file 1 [file Supplementary_file_1.docx]

Appendix A

Big Health Status Questionnaire for Primary and Secondary School Students

Dear student,

Hello! This survey aims to comprehensively understand students’ health and development status in order to help schools and educational authorities create better learning and living environments for you. Your responses are very important and will help us better understand students’ needs.

We sincerely promise that this questionnaire is anonymous. All information will be kept strictly confidential and used only for research purposes. There are no right or wrong answers. Please answer honestly based on your actual situation and feelings. The questionnaire will take approximately 15 minutes to complete.

Instructions: Please first complete your basic information. The questionnaire consists of four sections. Carefully read the instructions for each section and select the option that best describes your actual situation.

Please complete the questionnaire independently. If you do not understand any question, you may ask your teacher for clarification.

Part I: Basic Information

Educational level:

□ Primary school

□ Junior high school

□ Senior high school

Grade: ______

Gender:

□ Male

□ Female

Boarding status:

□ Boarding student

□ Non-boarding student

# Part II: Physical Health Status

Instruction: This section aims to understand your physical condition, lifestyle habits, and related health skills. Healthy lifestyle habits are the foundation of physical well-being.

Please indicate how well each statement describes you.

Response scale:

1 = Strongly disagree
2 = Disagree
3 = Neutral
4 = Agree
5 = Strongly agree

## **Section 1: Physical Health**

| Item Code | Statement |
| --- | --- |
| Health Knowledge | |
| A1 | I understand the importance of balanced nutrition, sufficient sleep, and regular exercise for maintaining physical health. |
| A2 | I am aware of the health risks associated with unhealthy eating habits and excessive use of electronic devices. |
| A3 | During the past week, I engaged in at least one hour of moderate to vigorous physical activity per day. |
| A4 | I maintain regular sleep habits and obtain sufficient sleep appropriate for my age. |
| A5 | I maintain good personal hygiene habits, such as brushing my teeth regularly and washing my hands properly. |
| A6 | I am able to limit my recreational screen time to an appropriate duration. |
| A7 | I know how to perform proper warm-up exercises and prevent injuries during physical activity. |
| A8 | I understand basic methods for managing minor sports injuries. |
| Single-item self-reported health-status variables (Please fill in based on recent physical examination or self-assessment)​ | |
| A9 | My vision condition (whether nearsighted and to what extent): □ No nearsightness □ Mild nearsightness □ Moderate nearsightness □ Severe nearsightness |
| A10 | How I feel about my body shape: □ Underweight □ Normal □ Overweight or obese |

## **Section 2: Psychological Health**

| Item Code | Statement |
| --- | --- |
| B1 | I generally experience stable and positive emotional states. |
| B2 | I am able to regulate my emotions effectively when experiencing negative feelings. |
| B3 | I am able to cope effectively with academic and life-related stress. |
| B4 | I am willing to seek help from others when facing difficulties. |
| B5 | I have confidence in my ability to overcome challenges. |
| B6 | I am aware of available psychological support resources. |
| B7 | I am generally satisfied with my life. |

## **Section 3: Social and Moral Health**

| Item Code | Statement |
| --- | --- |
| C1 | I am able to establish and maintain positive relationships with others. |
| C2 | I am willing to cooperate with others in group activities. |
| C3 | I am able to resolve conflicts through communication. |
| C4 | I feel respected and accepted in my school environment. |
| C5 | I believe honesty and rule-following are important. |
| C6 | I am willing to stand up for fairness and justice. |
| C7 | I respect individuals with different backgrounds and viewpoints. |
| C8 | I behave ethically even without external supervision. |
| C9 | I am willing to help others. |

## **Section 4: Environmental Health**

| Item Code | Statement |
| --- | --- |
| D1 | My family provides emotional support and encouragement. |
| D2 | My school provides sufficient opportunities for physical and social activities. |
| D3 | Teachers provide support when I encounter difficulties. |
| D4 | My school environment is positive and supportive. |
| D5 | My community environment is safe and supportive. |
